# The Value of Rare Genetic Variation in the Prediction of Common Obesity in European Ancestry Populations

**DOI:** 10.3389/fendo.2022.863893

**Published:** 2022-05-03

**Authors:** Zhe Wang, Shing Wan Choi, Nathalie Chami, Eric Boerwinkle, Myriam Fornage, Susan Redline, Joshua C. Bis, Jennifer A. Brody, Bruce M. Psaty, Wonji Kim, Merry-Lynn N. McDonald, Elizabeth A. Regan, Edwin K. Silverman, Ching-Ti Liu, Ramachandran S. Vasan, Rita R. Kalyani, Rasika A. Mathias, Lisa R. Yanek, Donna K. Arnett, Anne E. Justice, Kari E. North, Robert Kaplan, Susan R. Heckbert, Mariza de Andrade, Xiuqing Guo, Leslie A. Lange, Stephen S. Rich, Jerome I. Rotter, Patrick T. Ellinor, Steven A. Lubitz, John Blangero, M. Benjamin Shoemaker, Dawood Darbar, Mark T. Gladwin, Christine M. Albert, Daniel I. Chasman, Rebecca D. Jackson, Charles Kooperberg, Alexander P. Reiner, Paul F. O’Reilly, Ruth J. F. Loos

**Affiliations:** ^1^ The Charles Bronfman Institute for Personalized Medicine, Icahn School of Medicine at Mount Sinai, New York, NY, United States; ^2^ The Mindich Child Health and Development Institute, Icahn School of Medicine at Mount Sinai, New York, NY, United States; ^3^ Department of Genetics and Genomic Sciences, Icahn School of Medicine, Mount Sinai, New York, NY, United States; ^4^ Human Genetics Center, Department of Epidemiology, Human Genetics and Environmental Sciences, School of Public Health, The University of Texas Health Science Center at Houston, Houston, TX, United States; ^5^ Human Genome Sequencing Center, Baylor College of Medicine, Houston, TX, United States; ^6^ Brown Foundation Institute of Molecular Medicine, University of Texas Health Science Center at Houston, Houston, TX, United States; ^7^ Division of Sleep Medicine, Department of Medicine, Brigham and Women’s Hospital, Boston, MA, United States; ^8^ Department of Medicine, Beth Israel Deaconess Medical Center, Harvard Medical School, Boston, MA, United States; ^9^ Cardiovascular Health Research Unit, Department of Medicine, University of Washington, Seattle, WA, United States; ^10^ Department of Epidemiology, University of Washington, Seattle, WA, United States; ^11^ Channing Division of Network Medicine, Brigham and Women’s Hospital, Boston, MA, United States; ^12^ Division of Pulmonary, Allergy and Critical Care Medicine, Department of Medicine, University of Alabama at Birmingham, Birmingham, AL, United States; ^13^ Division of Rheumatology, Department of Medicine, National Jewish Health, Denver, CO, United States; ^14^ Channing Division of Network Medicine, Department of Medicine, Brigham and Women’s Hospital, Boston, MA, United States; ^15^ Department of Medicine, Harvard Medical School, Boston, MA, United States; ^16^ Department of Biostatistics, Boston University School of Public Health, Boston, MA, United States; ^17^ National Heart, Lung and Blood Institute’s and Boston University’s Framingham Heart Study, Framingham, MA, United States; ^18^ Section of Preventive Medicine and Epidemiology, Evans Department of Medicine, Boston University School of Medicine, Boston, MA, United States; ^19^ Whitaker Cardiovascular Institute and Cardiology Section, Evans Department of Medicine, Boston University School of Medicine, Boston, MA, United States; ^20^ Department of Medicine, Johns Hopkins University School of Medicine, Baltimore, MD, United States; ^21^ College of Public Health, University of Kentucky, Lexington, KY, United States; ^22^ Department of Population Health Services, Geisinger Health, Danville, PA, United States; ^23^ Department of Epidemiology, University of North Carolina at Chapel Hill, Chapel Hill, NC, United States; ^24^ Department of Epidemiology and Population Health, Albert Einstein College of Medicine, Bronx, NY, United States; ^25^ Kaiser Permanente Washington Health Research Institute, Seattle, WA, United States; ^26^ Division of Biomedical Statistics and Informatics, Mayo Clinic, Rochester, MN, United States; ^27^ The Institute for Translational Genomics and Population Sciences, Department of Pediatrics, The Lundquist Institute for Biomedical Innovation at Harbor-UCLA Medical Center, Torrance, CA, United States; ^28^ Division of Biomedical Informatics and Personalized Medicine, Department of Medicine, University of Colorado Anchutz Medical Camus, Aurora, CA, United States; ^29^ Center for Public Health Genomics, University of Virginia, Charlottesville, VA, United States; ^30^ Cardiovascular Disease Initiative, The Broad Institute of MIT and Harvard, Cambridge, MA, United States; ^31^ Cardiovascular Research Center, Massachusetts General Hospital, Boston, MA, United States; ^32^ Department of Human Genetics and South Texas Diabetes and Obesity Institute, University of Texas Rio Grande Valley School of Medicine, Brownsville, TX, United States; ^33^ Departments of Medicine, Pharmacology, and Biomedical Informatics, Vanderbilt University Medical Center, Nashville, TN, United States; ^34^ Division of Cardiology, University of Illinois at Chicago, Chicago, IL, United States; ^35^ Department of Medicine, University of Pittsburgh School of Medicine, Pittsburgh, PA, United States; ^36^ Department of Cardiology, Cedars-Sinai Medical Center, Los Angeles, CA, United States; ^37^ Division of Preventive Medicine, Brigham and Women’s Hospital, Boston, MA, United States; ^38^ Department of Medicine, Division of Endocrinology, Diabetes and Metabolism, The Ohio State University, Columbus, OH, United States; ^39^ Division of Public Health Sciences, Fred Hutchinson Cancer Research Center, Seattle, WA, United States; ^40^ Novo Nordisk Foundation Center for Basic Metabolic Research, Faculty of Health and Medical Sciences, University of Copenhagen, Copenhagen, Denmark

**Keywords:** polygenic risk score, rare variants, obesity risk, burden score, PRS-CS, lassosum, C+T, BMI - body mass index

## Abstract

Polygenic risk scores (PRSs) aggregate the effects of genetic variants across the genome and are used to predict risk of complex diseases, such as obesity. Current PRSs only include common variants (minor allele frequency (MAF) ≥1%), whereas the contribution of rare variants in PRSs to predict disease remains unknown. Here, we examine whether augmenting the standard common variant PRS (PRS_common_) with a rare variant PRS (PRS_rare_) improves prediction of obesity. We used genome-wide genotyped and imputed data on 451,145 European-ancestry participants of the UK Biobank, as well as whole exome sequencing (WES) data on 184,385 participants. We performed single variant analyses (for both common and rare variants) and gene-based analyses (for rare variants) for association with BMI (kg/m^2^), obesity (BMI ≥ 30 kg/m^2^), and extreme obesity (BMI ≥ 40 kg/m^2^). We built PRSs_common_ and PRSs_rare_ using a range of methods (Clumping+Thresholding [C+T], PRS-CS, lassosum, gene-burden test). We selected the best-performing PRSs and assessed their performance in 36,757 European-ancestry unrelated participants with whole genome sequencing (WGS) data from the Trans-Omics for Precision Medicine (TOPMed) program. The best-performing PRS_common_ explained 10.1% of variation in BMI, and 18.3% and 22.5% of the susceptibility to obesity and extreme obesity, respectively, whereas the best-performing PRS_rare_ explained 1.49%, and 2.97% and 3.68%, respectively. The PRS_rare_ was associated with an increased risk of obesity and extreme obesity (OR_obesity_ = 1.37 per SD_PRS_, *P*_obesity_ = 1.7x10^-85^; OR_extremeobesity_ = 1.55 per SD_PRS_, *P*_extremeobesity_ = 3.8x10^-40^), which was attenuated, after adjusting for PRS_common_ (OR_obesity_ = 1.08 per SD_PRS_, *P*_obesity_ = 9.8x10^-6^; OR_extremeobesity_= 1.09 per SD_PRS_, *P*_extremeobesity_ = 0.02). When PRS_rare_ and PRS_common_ are combined, the increase in explained variance attributed to PRS_rare_ was small (incremental Nagelkerke R^2^ = 0.24% for obesity and 0.51% for extreme obesity). Consistently, combining PRS_rare_ to PRS_common_ provided little improvement to the prediction of obesity (PRS_rare_ AUC = 0.591; PRS_common_ AUC = 0.708; PRS_combined_ AUC = 0.710). In summary, while rare variants show convincing association with BMI, obesity and extreme obesity, the PRS_rare_ provides limited improvement over PRS_common_ in the prediction of obesity risk, based on these large populations.

## Introduction

With an estimated prevalence of 12% among adults worldwide and up to 42% in the US (1, 2), obesity is a growing epidemic, causing major public health concerns (1, 3). Risk prediction and early prevention of weight gain is key to reducing the personal and global burden of obesity and its comorbidities (4). Developing obesity across the lifespan is the result of an interaction between environmental and innate biological factors, encoded by our genomes. Twin and family studies have reported heritability estimates of obesity that range between 40 - 70% (5).

In the past 15 years, genome-wide association studies (GWAS) have identified thousands of variants associated with obesity-related traits (6). Polygenic risk scores (PRSs), which are based on GWAS summary statistics, represent an individual’s overall genetic predisposition to obesity. In recent years, PRSs have been studied for their use in the prediction of future obesity and the identification of individuals at risk of obesity early on in life (7). The promise is that accurate estimation of people’s genetic predisposition would allow more targeted lifestyle intervention for those at risk. However, current PRSs, which are based on traditional GWAS, have been shown to be suboptimal, with unsolved challenges remaining (8). For example, existing methods to develop PRSs only include common variants (MAF ≥ 1%), they explain little of the variation (< 10%) in BMI and, thus, have limited ability to predict obesity (7, 9). There is a pressing need to incorporate rare variants (MAF < 1%), which have been shown to capture a proportion of the ‘missing heritability’ (10), and are currently not considered in the PRS construction.

Including rare variants in the PRS may improve the accuracy with which we estimate individuals’ genetic predisposition. Because of the large sample size of studies, such as the UK Biobank, association summary statistics for rare variants (0.1% ≤ MAF < 1%) can be assessed by single variant testing (11). However, for ultra-rare variants (MAF < 0.1%), which occur by definition very infrequently in the population, even current large-scale studies are not large enough to study their individual effects (12). The accuracy of the PRS depends largely on the power of the discovery GWAS summary statistics (13). Therefore, aggregating ultra-rare variants in genes, based on their predicted functional consequences, offers a potentially powerful complementary approach to the single variant testing (14) and subsequently, building rare variant PRSs.

The aim of our study is to leverage sequencing data from the UK Biobank and the Trans-Omics for Precision Medicine (TOPMed) program to build obesity PRSs that use rare variants (PRSs_rare_) and test their associations with obesity and extreme obesity. In addition, we will test the predictive power of PRSs_rare_ for obesity outcomes alone or in combination PRSs_common_.

## Materials and Methods

### Study Design

**Figure 1:**
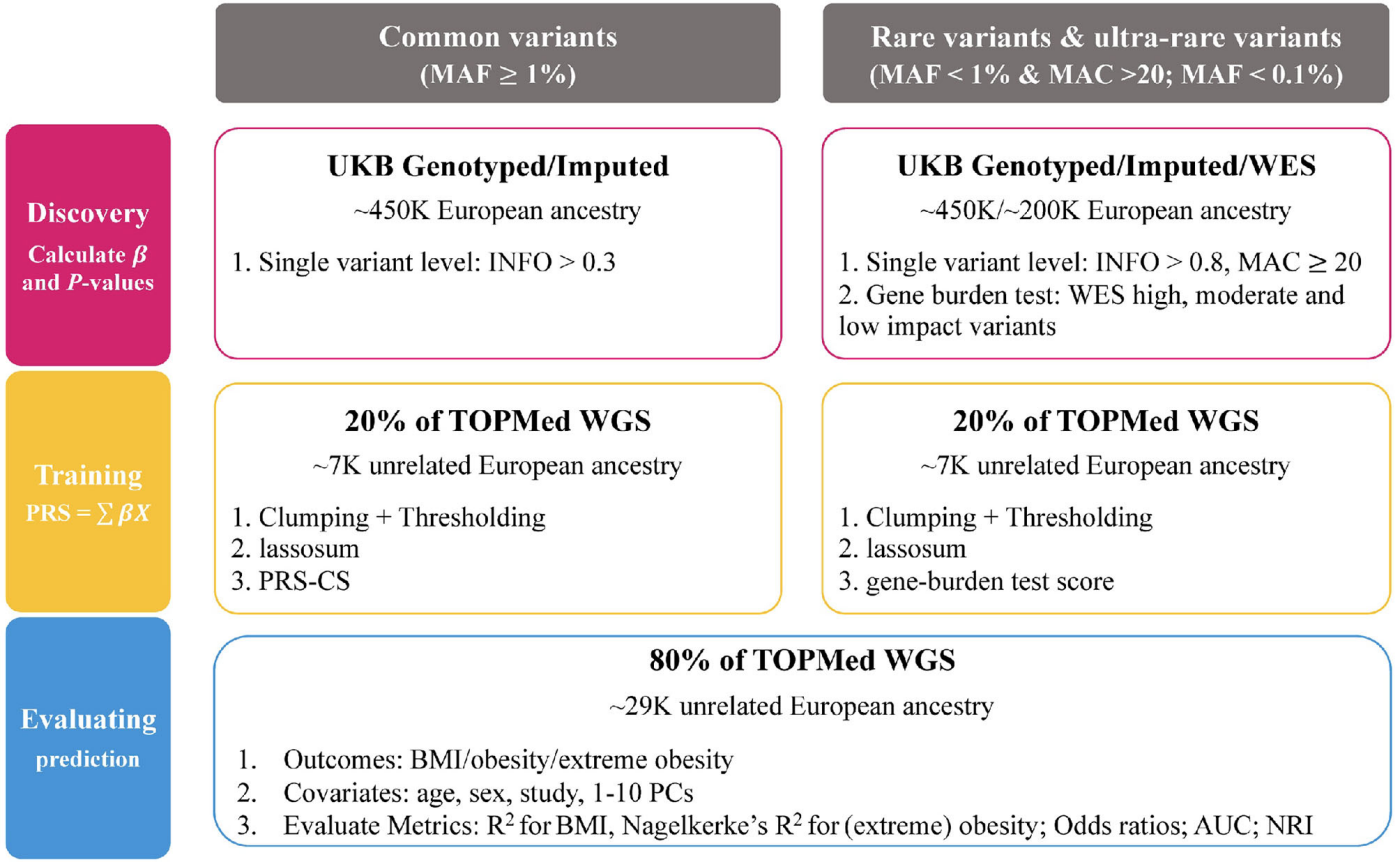
Overview of the study framework.

We built and tested PRSs from common variants (MAF ≥ 1%), rare variants (MAF < 1%) and ultra-rare variants (MAF < 0.1%) for three traits; BMI, obesity and extreme obesity. We used data from the UK Biobank to conduct single variant GWAS analyses and gene burden analyses (ultra-rare variants). Then, the GWAS summary statistics, calculated using the UK Biobank data, were used to build PRSs for which we tested the predictive performance in the TOPMed program (**Figure 1**).

### Study Populations

#### UK Biobank

All GWAS analyses were performed using data of the UK Biobank, a prospective cohort study with extensive genetic and phenotypic data collected in approximately 500,000 individuals, aged between 40–69 years (11). Briefly, participants were enrolled from 2006 to 2010 at one of 22 assessment centers across the UK to provide baseline information, physical measures, and biological samples according to standardized procedures (11, 15). All participants provided written informed consent. We restricted analyses to individuals of European ancestry (described in detail below), excluded individuals who underwent weight loss surgery before recruitment and women who were pregnant at the time of recruitment. Data for 451,145 individuals was available for analyses.

#### TOPMed

For constructing and testing the PRS, we used data from 22 parent studies of the TOPMed program (**Supplementary Table 1**). We restricted analyses to 43,251 individuals of European ancestry that have cleaned phenotype data (described in detail below) and Whole Genome Sequencing (WGS) data. We removed one individual from each related pair (N_excl_ = 6,494; genetic relatedness ≥.0625). In addition, we removed Data for a total of 36,757 individuals were available for analyses (**Supplementary Table 1**).

### Phenotype Definitions

#### UK Biobank

Height and weight, used to calculate BMI as weight (kg) divided by height squared (m^2^), were collected at the baseline visit. BMI was used to categorize individuals with underweight (BMI < 18.5 kg/m^2^), normal weight (18.5 kg/m^2^ ≤ BMI < 25 kg/m^2^), overweight (25 kg/m^2^ ≤ BMI < 30 kg/m^2^), obesity (BMI ≥ 30 kg/m^2^) or extreme obesity (BMI ≥ 40 kg/m^2^). More details can be found elsewhere (11, 15).

#### TOPMed

Data on height and weight, used to calculate BMI, were harmonized across studies by the TOPMed Anthropometry Working Group. BMI was calculated based on weight and height measurements, collected from the participating studies. We excluded individuals with known pregnancy at measurement, with implausibly high BMI values (> 100 kg/m^2^), and those < 18 years old. In the presence of duplicated samples, the sample with the highest sequencing depth was retained.

### Genotyping, Imputation and Sequencing Data

#### UK Biobank

All UK Biobank participants were genotyped using the UK Biobank Axiom Array. More than 800,000 variants were directly genotyped and > 90 million variants were imputed, using the Haplotype Reference Consortium or UK10K + 1000G reference panels (11). Variants with imputation INFO score of ≥ 0.3 for common (MAF ≥ 1%), and imputation INFO score of ≥ 0.8 for rare variants (MAF < 1%) were included in analyses.

We identified individuals of European ancestry based on their genetic information, using k-means clustering. First, we calculated principal components and their loadings for 488,377 genotyped UK Biobank participants based on the intersection of ~121,000 variants after quality control and 1000G Phase 3v5 reference panel. Reference ancestries are 504 European (EUR), 347 American Admixed (AMR), 661 African (AFR), 504 East Asian (EAS) and 489 South Asian (SAS) samples (overall 2504). We projected the 1000G reference panel dataset based on the calculated PCA loadings from UK Biobank. We then used k-means clustering with a pre-specified amount of 4 clusters to the UK Biobank PCA and the projected 1000G reference panel dataset. Individuals that clustered within the EUR individual cluster from the 1000G reference panel were assigned as individuals of European ancestry (N = 453,812). Because PRSs based on current methods generalize poorly across other ancestries, and because of the smaller sample sizes of non-European ancestry population, we performed analyses only in European ancestry populations.

In addition to the genotyped and imputed data, we used data of the first release of exome sequencing (N=184,385). The approach used to perform exome sequencing and quality control is described in detail elsewhere (16, 17). We annotated variants using Variant Effect Predictor (VEP) v104.3 with genome build GRCh38 (18).

#### TOPMed

WGS, targeting a mean depth of >30X coverage, was performed at seven different Sequencing Centers. For this study, we used WGS data from Freeze 8 release (19). Information about genome sequencing, variant calling, and quality control procedures can be accessed through the TOPMed website (20). The genetic relationship was estimated using the PC-Relate algorithm (21). We removed one from each pair of the individuals with genetic relationship closer than 3rd degree (≥.0625) of relatedness (21).

Population groups in TOPMed were based on a combination of participants’ self-reported race/ethnicity and genetic ancestry represented by PCs. When participants’ self-reported race/ethnicity values were “Other”, “Multiple” or missing, the HARE method was used to classify individuals into “Asian”, “Black”, “White”, or “Hispanic/Latino” subgroups using the first nine PC-AiR PCs (22). For this project, we limited our analyses to those either self-identified as “White” or they had overall genetic ancestry that closely resembled groups of European ancestry (HARE strata classified as ‘White”).

### Genome-Wide Association Testing: Single Variant and Gene Burden Tests in UK Biobank

BMI residuals were generated in men and women separately, adjusting for age, age^2^, and the first 10 genetic principal components (PCs). Residuals underwent inverse normal transformation, to achieve a normal distribution with a mean of 0 and a standard deviation of 1.

#### Single Variant Association Testing

Association analyses of the inverse normal BMI residuals, obesity, and extreme obesity were carried out using a (generalized) linear mixed-model approach in BOLT-LMM (23) and REGENIE (24). Models were adjusted for age, age^2^, sex and first 10 PCs for obesity and extreme obesity. For all single variant association testing, variants with a minor allele count of ≤20 were excluded. We performed single variant association testing using [1] genotyped and imputed variants, and [2] WES data, separately.

#### Gene Burden Testing

**Figure 2:**
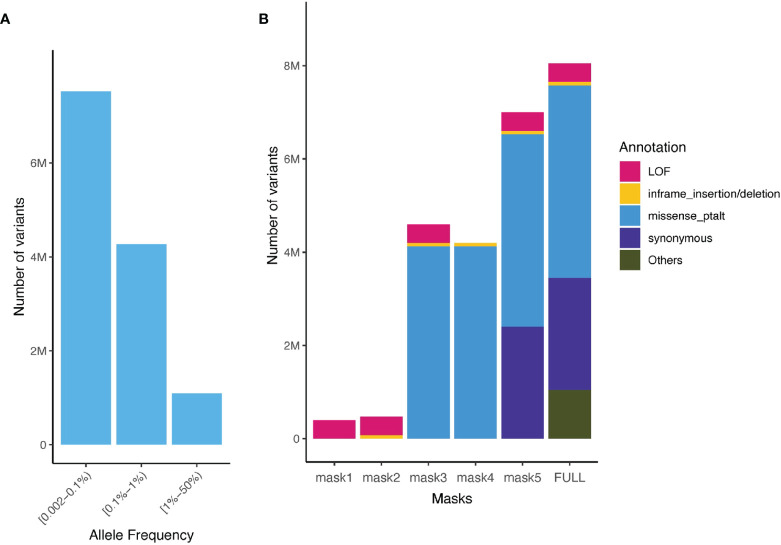
Allele frequency spectrum of imputed variants and number of aggregated sequenced variants captured in the UK Biobank and the TOPMed. **(A)** Minor allele frequency spectrum of imputed variants present in the UK Biobank (rare variants imputation INFO ≥ 0.8, common Hapmap3 variants imputation INFO ≥ 0.3) and TOPMed; **(B)** Number of variants for different functional class of variants and masks (aggregation model) in the UK Biobank WES ultra-rare variants (MAF < 0.1%).

We aggregated ultra-rare variants (MAF < 0.1%) from the WES data for gene burden testing. For each gene, we considered five categories of masks (i.e. variant sets considered in burden test): [M1] a strict burden of rare loss-of-function (LoF) variants (i.e. splice_acceptor, splice_donor, stop_gained, frameshift, stop_lost, and start_lost), [M2] a permissive burden of rare LoF variants and inframe indels, [M3] a more permissive burden of all high and moderate impact rare variants (including LoF, inframe indels, and missense variants) [M4] moderate impact variants (inframe indels and missense variants), and [M5] high, moderate and low impact variants (LoF, inframe indels, missense and synonymous variants, **Figure 2**). We aggregated MAF ≤ 0.1% variants for each of these masks, that is up to 5 burden tests per gene.

### Polygenic Risk Score Derivation in TOPMed

Based on the single variant association testing and gene burden testing results in UK Biobank, we generated PRSs_common_ and PRSs_rare_ using three different approaches (PRS_common_: Clumping + Thresholding [C+T], PRS-CS (18), lassosum (25); PRS_rare_: C+T, lassosum, gene-burden test) in 36,757 unrelated individuals of European ancestry of TOPMed. Summary statistics from GWAS of the UK Biobank were filtered for variants present in TOPMed (**Figure 2**).

C+T denotes the Linkage Disequilibrium (LD) clumping and *P* value thresholding method, which was conducted using the PRSice-2 software (26). For clumping, we used the entire sample of 36,757 unrelated individuals of European ancestry as the reference panel for LD and set clumping parameters to R^2 =^ 0.2, 0.5 and 0.8, with each region being 250kb in size. We varied the *P* value thresholds from 5x10^-5^ to 0.8, with a step-wise increase of 1x10^-4^. The C +T method was used to build both PRS_common_ and PRS_rare_.

PRS-CS is a Bayesian method that infers the posterior mean effect size of each variant using GWAS summary statistics and external LD (27), but is distinct from previous methods by placing a continuous shrinkage (CS) prior on the variant effect sizes (27). A 1000G LD reference panel for European ancestry populations was provided by the developers. We followed the PRS-CS author recommended protocol by removing ambiguous A/T or G/C variants and restricting to common variants (MAF ≥ 1%) included in HapMap3. Therefore, this method was used only to build PRS_common_. We considered the shrinkage prior (phi = 1x10^-3^, 1x10^-4^) and the PRS-CS auto option, which allows the software to learn the continuous shrinkage prior from the data.

lassosum is an approach that uses penalized regression on summary statistics and accounts for LD using an external reference panel or target sample to produce more accurate weights for building PRSs (25). To accurately assess the LD – particularly important for rare variants – we used the entire sample of 36,757 unrelated individuals of European ancestry TOPMed as the reference panel. lassosum’s model parameters (s, the shrinkage parameter: 0.2, 0.5, 0.9 and 1; and λ, the penalty parameter: varied from 0.001 to 0.1) were tuned. We applied the lassosum method to common and rare variants separately to build PRS_common_ and PRS_rare_.

Lastly, we built ultra-rare variant burden scores using the gene burden test results from the UK Biobank. For each of the five masks, we tested the following *P* value threshold of gene burden tests; P = 0.05, 0.001, 0.0001, 10^-5^, and 2.8x10^-6^ (i.e. exome-wide significance level). For assigning weights to variants within each gene, we tested two methods: 1) a simple method, which assigned the same weights to all variants in the same mask (i.e. using the aggregate effect size estimated from LoF (mask1) gene A in UK Biobank to the LoF (mask1) variants in gene A in the TOPMed samples); 2) a nested method, which assigned a weight to each variant equal to the aggregate effect size of variants with annotation at least as severe as the variant (**Supplementary Figure 1** provides an example to illustrate the nested method).

For each individual in the testing sets (TOPMed), PRSs were calculated as the sum of the dosages multiplied by the given weight at each variant. Taken together, we generated six sets of PRSs (PRS_common-C+T_, PRS_common-lassosum_, PRS_common-PRS-CS_, PRS_rare-C+T_, PRS_rare-lassosum_, and PRS_rare-burden_) for each trait (BMI, obesity and extreme obesity) using the different methods under a range of tuning parameters.

### Statistical Analyses

BMI in TOPMed was inverse rank normalized, in men and women separately. We split unrelated individuals in TOPMed by randomly selecting 20% for PRS training (N=7,433, tuning parameter and selecting the best performing PRS) and 80% for evaluation (N=29,324, validating R^2^ and predicting performance). For each PRS method applied, we calculated adjusted R^2^ values for BMI and Nagelkerke R^2^ values for (extreme) obesity. Models were adjusted for age, sex, the first ten PCs and study. 95% confidence intervals were calculated using bootstrapping. We selected the best-performing PRS for each method and PRS combination (i.e. the largest variance explained (adjusted R^2^ values or Nagelkerke R^2^), resulting in six best-performing PRSs in total (one for each from PRS_common-C+T_, PRS_common-lassosum_, PRS_common-PRS-CS_, PRS_rare-C+T_, PRS_rare-lassosum_, and PRS_rare-burden_).

In the 80% withheld TOPMed individuals, we tested the association between each PRS and obesity/extreme obesity status using logistic regression. The best-performing PRS_common_ and PRS_rare_ across multiple methods were then combined to study the joint effects of PRS_common_ and PRS_rare_ to predict obesity. To evaluate the prediction performance of PRS_rare_, we calculated the area under the receiver operator curve (AUC) in a Cox regression model with the obesity/extreme obesity status as the outcome. We also assessed the net reclassification index (NRI) and the Integrated Discrimination Increment (IDI), which evaluated the model improvement in discrimination and reclassification.

## Results

### Best-Performing Polygenic Risk Scores Based on Common Variants (PRSscommon)

**Figure 3:**
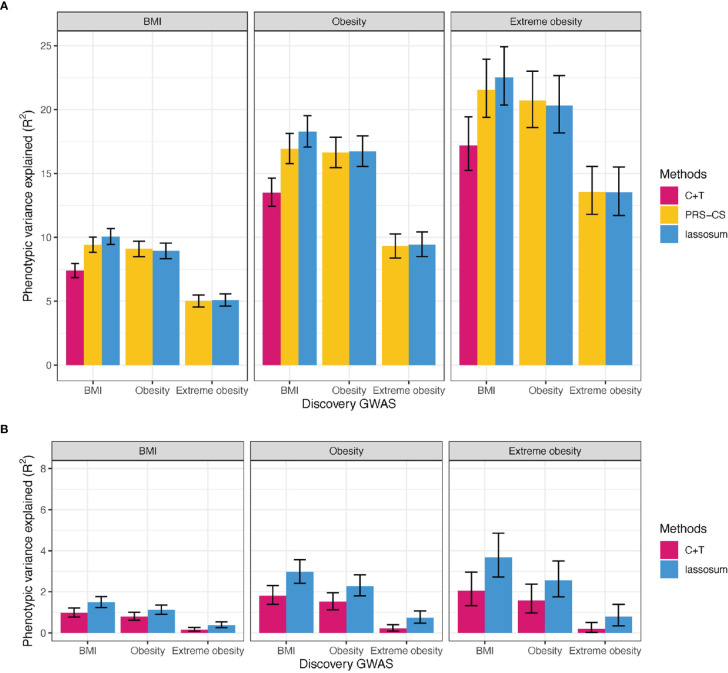
Variance explained by PRS for BMI, obesity, and extreme obesity in BMI, obesity and extreme obesity. **(A)** PRScommon **(B)** PRSrare, We reported adjusted R^2^ for BMI, Nagelkerke’s R^2^ for (extreme) obesity on top of covariates including age, sex, study and PCs. C+T: Clumping and Thresholding method. Error bars indicates 95% CI.

Using BMI-GWAS summary statistics derived in the UK Biobank (**Supplementary Figure 2**), the PRS_common_ built with the lassosum method (**Supplementary Table 2** and **Figure 3**) explained the most variation in BMI (R^2^ = 10.1%, 95% CI = 9.4-10.7%).

Similarly, the best-performing PRSs_common_ based on summary statistics of obesity and extreme obesity GWASs, was built using lassosum (Nagelkerke R^2^ = 16.7% for obesity and 20.7% for extreme obesity, **Supplementary Table 2** and **Figure 3**). Of interest is that that the PRS_common_ based on BMI-GWAS summary statistics explained more of the variation in (extreme) obesity (Nagelkerke R^2^ = 18.3% for obesity and 22.5% for extreme obesity) than the PRS_common_ based on (extreme) obesity GWAS summary statistics (**Figure 3**). This likely reflects the relatively higher power of the BMI GWAS.

### Best-Performing Polygenic Risk Scores Based on Rare Variants (PRSsrare) at Single Variant Level

The best-performing PRS_rare_ for BMI was built using the lassosum method, based on BMI-GWAS summary statistics, explaining 1.49% of variation in BMI (95% CI = 1.23-1.77%, **Supplementary Table 2** and **Figure 3**). Consistent with our observations for the PRSs_common_, a PRS_rare_ based on BMI-GWAS summary statistics explained more of the variance for (extreme) obesity liability (Nagelkerke R^2^ = 2.97% for obesity and 3.68% for extreme obesity) than a PRS_rare_ based on (extreme) obesity GWAS (Nagelkerke R^2^ = 2.28% for obesity and 2.55% for extreme obesity) (**Figure 3**).

### Best-Performing Polygenic Risk Score Based on Ultra-Rare Variants (PRSrare-Burden) Using Gene Burden Score

Aggregating variants using mask1 (LoF variants) with an association significance of *P* < 2.8x10^-6^ resulted in the best-performing PRS*_rare-burden_*, explaining a mere 0.03% (95%CI = 0.002-0.08%) of variation in BMI (**Methods**, **Supplementary Figure 3** and **Supplementary Figure 4**). However, this PRS*_rare-burden_* aggregated LoF variants in only two genes (*MC4R* and *UBN2*) and identified 2,957 individuals (8% of the TOPMed population) with non-zero values of the score (**Supplementary Figure 4**).

We repeated the gene burden score approach using summary statistics of obesity and extreme obesity (**Supplementary Figure 5**), yielding slightly improved results than for a PRS_rare-burden_ based on BMI summary statistics. Mask3, which aggregates variants in genes that reached exome-wide significance—only *MC4R* meets this P-value threshold (*P* < 2.8x10^-6^)*—*provided the best-performing PRS_rare-burden_ score, explaining 0.08% of variation in obesity and 0.39% of variation in extreme obesity liability.

### Association of PRSscommon and PRSsrare With Risk of Obesity

We next tested the association of the best-performing PRSs (i.e. PRS_common-lassosum_ and PRS_rare-lassosum_ based on BMI-GWAS summary statistics and PRS_rare-burden_ based on obesity-GWAS summary statistics) with obesity outcome.

Each SD increase in the BMI-GWAS based PRS_rare-lassosum_ was associated with a 1.37 (*P* = 1.7x10^-85^) increase in the odds of obesity (**Supplementary Table 3**). Adding PRS_common-lassosum_ to the model substantially attenuated the association between PRS_rare-lassosum_ and risk of obesity (OR = 1.08 per SD, *P* = 9.8x10^-6^). This attenuation is likely due to the correlation between PRS_rare-lassosum_ and PRS_common-lassosum_ (r = 0.31). Each 0.1 increase in obesity-GWAS based PRS_rare-burden_ (range: 0 - 0.41) was associated with a 1.83 higher odds of obesity (*P* = 0.02). Adding the PRS_common-lassosum,_ (r = 0.008) and/or PRS_rare-lassosum_ (r=0.01) had little impact on the association (**Supplementary Table 3**). We observed a similar pattern for the PRSs’ associations with extreme obesity (**Supplementary Table 3**). Consistently, adding both PRS_rare-lassosum_ and PRS_rare-burden_ in addition to model with PRS_common_ was extremely small (incremental Nagelkerke R^2^ 0.24% for obesity and 0.51% for extreme obesity, **Supplementary Table 3**).

**Figure 4:**
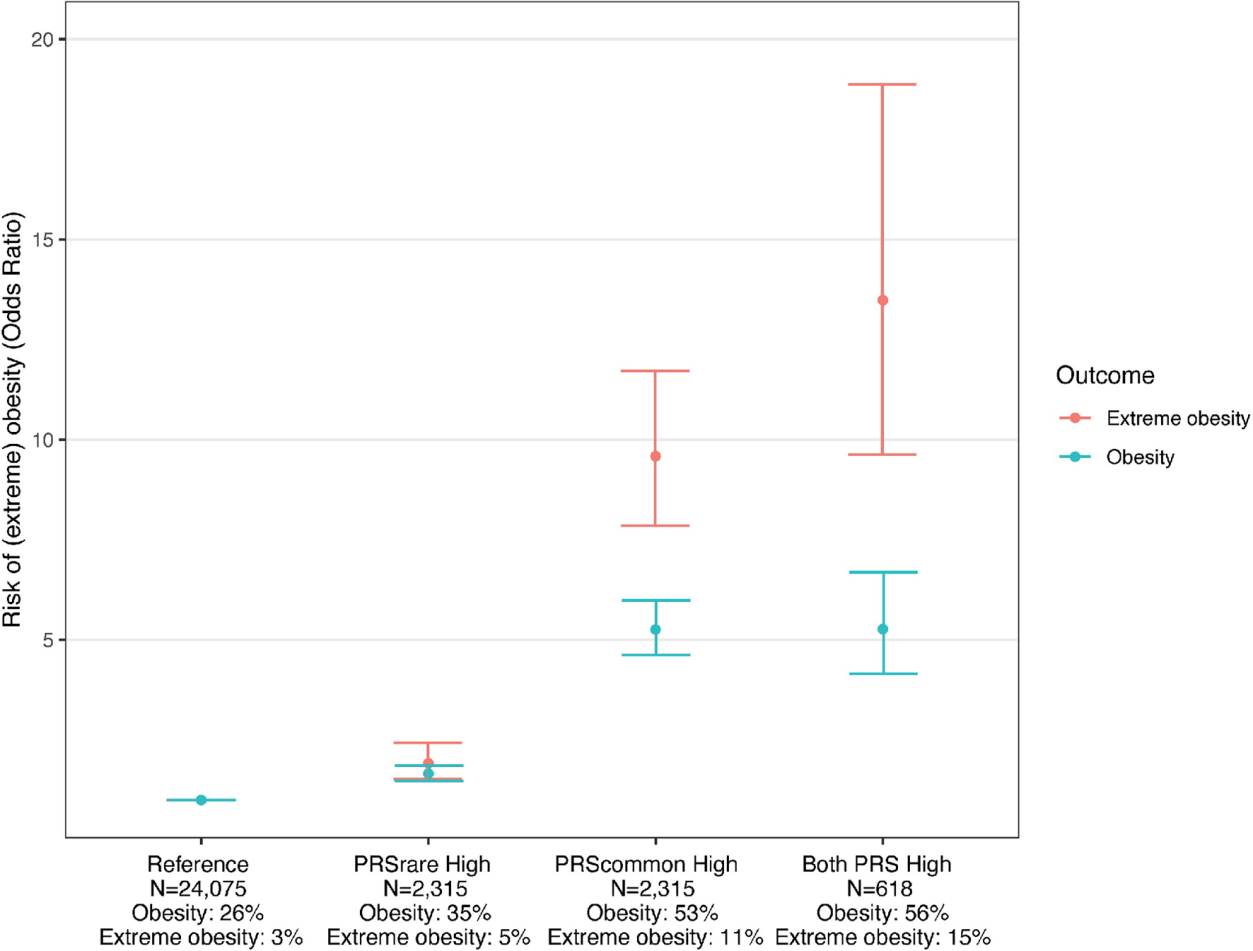
Risk of obesity among individuals with high PRSrare and PRScommon. Reference: deciles 1-9 of PRS_common_ and PRS_rare_, PRS_rare_ High: top decile of PRS_rare_, PRS_common_ High: top decile of PRS_common_, Both PRS High: top decile of PRS_common_ and PRS_rare_.

Using the PRS_common-lassosum_ and PRS_rare-lassosum_ to identify individuals at high risk of obesity (top PRS decile), we observe that, relative to the reference group (deciles 1-9), individuals in the top decile for both PRSs had the highest risk of obesity and extreme obesity (OR [95%CI] = 5.3 [4.2-6.7], 13.5 [9.6-18.9], respectively), as compared to individuals that were defined as high risk by only one of the two PRSs (**Figure 4**).

### Using PRScommon and PRSrare to Predict Common Obesity

**Figure 5:**
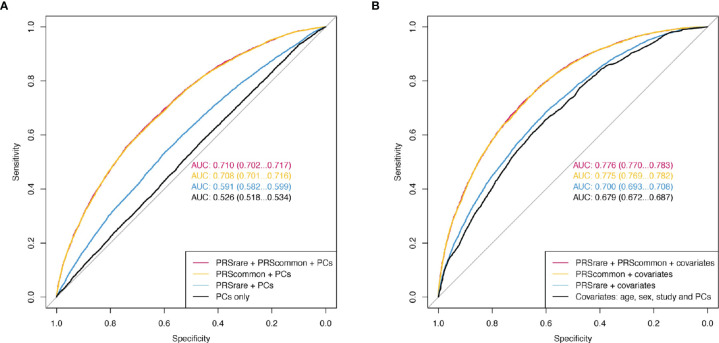
The receiver operating characteristic curve (ROC) of obesity. **(A)** Model only included PCs as baseline covariates. **(B)** Additionally included age, sex, and study. PRSrare includes PRS_rare-lassosum_ and PRS_rare-burden_.

Adding both PRS_rare-lassosum_ and PRS_rare-burden_ to PRS_common-lassosum_ in the prediction model did not improve the prediction of obesity (PRS_common_ only AUC [95%CI] 0.708 [0.701 – 0.716] *vs* all three PRSs 0.710 [0.702 – 0.717], **Figure 5**). Adding both PRS_rare-lassosum_ and PRS_rare-burden_ to a model with PRS_common-lassosum_ only slightly improved the discrimination of the model (IDI= 0.0014 [0.0008 - 0.0019], **Supplementary Table 4**). Knowledge of individuals’ PRS_rare-lassosum_ and PRS_rare-burden_, in addition to the PRS_common-lassosum_, would only reassign 0.9% of individuals to their appropriate risk category (NRI=0.9%; 95%CI= 0.49-1.32%; *P* = 2x10^-5^). Using extreme obesity as the outcome yielded similarly small improvements in predictive accuracy (**Supplementary Table 4**, **Supplementary Figure 6**).

## Discussion

In this study, we examined the contribution of rare variants to the polygenic prediction of obesity by leveraging data from 451,145 European-ancestry individuals in UK Biobank and 36,757 in TOPMed. We observed that PRSs_rare_ were associated with an increased risk of obesity and extreme obesity, partially independent of PRS_common_. Nevertheless, their explained variance (up to 1.49%) as well as predictive accuracy were small (AUC 0.591 for obesity and 0.630 for extreme obesity), and particularly limited when considered in combination with PRS_common_.

As PRSs are becoming a standard tools in translational research and clinical practice, there has been an increasing interest to study the role of rare variants, in addition to common ones, for a range of common diseases, such as breast cancer, prostate cancer, coronary artery disease (CAD) and obesity (28–31). Most previous studies that have reported on the contribution of rare variants studied the role of pathogenic variants in one or few high-penetrance genes and did not investigate their predictive accuracy at a population level (28, 29, 31). Consistent with our findings, though, these studies demonstrated that rare variants act—at least in part—independently from common variant PRSs and add to people’s polygenic susceptibility to disease (28, 29, 31). Thus, knowing an individuals’ PRS_rare_, in addition to PRS_common_, may contribute to identifying individuals at high risk of obesity. However, given the limited explained variance observed in our analyses, we expect that few individuals will indeed score high on both scores. Nevertheless, for these few individuals, knowing their high risk may be valuable.

Recently, a new framework was developed to aggregate rare variant burden into a rare variant PRS (30). As an example, a rare variant genetic risk score for CAD was built, using UK Biobank data. Similar to our findings for obesity and extreme obesity, a significant association of this PRS_rare_ with risk of CAD was observed, although the explained variation was only 0.1% of the population variance (30). We report a similar explained variance of 0.2% for obesity and 0.5% for extreme obesity. The reasons why the PRS_rare_’s explained variance is small, in particular in addition to the PRS_common_, are threefold. First, the PRS_rare_ was not completely independent from PRS_common_, even after including only non-overlapping variants. It is likely that the true causal (rare) variants were tagged by common variants in LD. Second, any new (rare) variant added to the PRS increases the PRS’ uncertainty due to statistical noise associated with estimating a new weight (32). The PRS_rare_ might have suffered more from this, as accurately estimating weights for rare variants requires larger sample size in general. Third, rare variants, although more likely to have larger effects (12), are too rare to explain much of the obesity epidemic in the general population.

Consistent with the low variance explained, the predictive power by the PRS_rare_ over that of the PRS_common_ was limited. The improvement in AUC for obesity (from 0.708 to 0.710) was negligible, although the AUC for the PRS_rare_ alone was up to 0.59. This supports our observation that the predictive power of the PRS_rare_ in part overlapped with that of the PRS_common_. So far, no other studies have reported on the contribution of PRS_rare,_ in the presence of PRS_common_.

In addition to using BMI summary statistics to build PRSs and test their predictive performance for obesity and extreme obesity, we built PRSs_common_ and PRSs_rare_ based on obesity and extreme obesity GWAS summary statistics. The PRS_common_ and PRS_rare_ based on BMI-GWAS summary statistics outperformed those based on obesity or extreme obesity GWAS summary statistics, which is in line with previous findings that PRS_common_ based on the full distribution explains a larger proportion of the variance than when based on the tails of the distribution (33). For the ultra-rare variants, the PRS_rare-burden_ based on obesity summary statistics performed better than the those based BMI-based summary statistics, which maybe be due to the role of ultra-rare variants in (extreme) obesity, but less in BMI. Our discovery GWASs were conducted in a relatively healthy and less deprived UK Biobank population (34), which may have limited our ability to capture the genetic contribution of rare variants for obesity and extreme obesity.

We acknowledged that our samples for analyses were restricted to one ancestry only. We focused our analyses on European-ancestry populations for which the most data are available. Because allele frequencies, LD patterns, and effect sizes, differ between ancestries, the accuracy of European-derived PRSs decays rapidly when applied to other ancestries (35). PRSs derived from other ancestries are currently underpowered because of relatively small sample sizes. As more data becomes available for other ancestries, both GWAS as well as sequencing data, the here described analyses should be performed to examine whether observation are generalizable across ancestries. Furthermore, we focused solely on obesity, a common multifactorial trait that is moderately heritable. While many complex traits have similar feature, we cannot guarantee that our observations can be extrapolated to other outcomes as the genetic architecture, explained variance from common variants, and contribution from rare pathogenic variants may differ (36).

Taken together, we demonstrate that while rare variants, aggregated in PRSs_rare_, have been shown to independently associate with obesity risk, they provide a minimal improvement in prediction accuracy over PRS_common_ in predicting obesity risk in the general population. Our findings cast an important light on the potential value of rare variants in the prediction of complex diseases, such as obesity.

## Data Availability Statement

Publicly available datasets were analyzed in this study. UK Biobank data can be found here: UK Biobank (https://www.ukbiobank.ac.uk/). All TOPMed data for each participating study can be accessed through dbGaP with the corresponding accession number listed in Acknowledgments.

## Ethics Statement

All phenotypic and genetic data were collected with approval from the Institutional Review Board with patient consent at each institution. This study was approved by the Institutional Review Board (IRB) of the Icahn School of Medicine at Mount Sinai in New York, New York.

## Author Contributions

Study concept and design: ZW and RL. Acquisition of cohort level data: EB, RL, ZW, NC, MF, SR, BP, JAB, JCB, ES, M-LM, ER, WK, RV, C-TL, RM, LY, RRK, DA, RK, KN, AJ, SH, MA, JR, XG, LL, SSR, PE, SL, JB, MS, DD, MG, CA, DC, CK, RJ, and AR. Statistical analysis: ZW and SC. Interpretation of data: ZW, PFO, and RL. Manuscript writing group: ZW, PFO, SC, and RL. Supervision: PFO and RL. All authors contributed to the article and approved the submitted version.

## Author Disclaimer

The views expressed in this manuscript are those of the authors and do not necessarily represent the views of the National Heart, Lung, and Blood Institute; the National Institutes of Health; or the U.S. Department of Health and Human Services.

## Conflict of Interest

BP serves on the Steering Committee of the Yale Open Data Access Project funded by Johnson & Johnson. PE has received sponsored research support from Bayer AG and from IBM Research and has also served on advisory boards or consulted for Bayer AG, Quest Diagnostics, MyoKardia and Novartis. SL receives sponsored research support from Bristol Myers Squibb/Pfizer, Bayer AG, Boehringer Ingelheim, Fitbit, and IBM, and has consulted for Bristol Myers Squibb/Pfizer, Blackstone Life Sciences, and Invitae. ES has received grant support from GSK and Bayer.

The handling editor declared a past co-authorship with one of the authors RL.

The remaining authors declare that the research was conducted in the absence of any commercial or financial relationships that could be construed as a potential conflict of interest.

## Publisher’s Note

All claims expressed in this article are solely those of the authors and do not necessarily represent those of their affiliated organizations, or those of the publisher, the editors and the reviewers. Any product that may be evaluated in this article, or claim that may be made by its manufacturer, is not guaranteed or endorsed by the publisher.
